# Sandwich graft technique outcomes in medium and large size nasal septal perforations

**DOI:** 10.1016/j.bjorl.2020.12.018

**Published:** 2021-02-13

**Authors:** Serdar Özer, Ahmet Emre Süslü, Taner Yılmaz, Tevfik Metin Önerci

**Affiliations:** Hacettepe University, Faculty of Medicine, Department of Otorhinolaryngology-Head & Neck Surgery, Ankara, Turkey

**Keywords:** Interposition graft, Nasal septal perforation, Nasal septum, Sandwich technique, Septal perforation repair

## Abstract

**Introduction:**

Surgical treatment of medium and large sized nasal septal perforation is challenging. Techniques with and without interposition grafts are used.

**Objective:**

The aim of this study is to explain how we apply the sandwich graft technique that we use in medium and large nasal septal perforations as well as to present the results.

**Methods:**

We retrospectively reviewed the patients who were operated with the sandwich graft technique between January 2014 to December 2018 and followed up for at least 6 months. The demographic data, symptom scores, examination, and surgical findings of the patients were taken from the hospital records. Surgical outcomes were presented according to both perforation etiologies (idiopathic or iatrogenic) and sizes (Group A: < 2 cm, Group B: ≥ 2 cm).

**Results:**

We reviewed 52 cases and 56 surgeries. The average diameter of the perforations was 19.2 mm. The success rate after initial surgeries was 84.6% (44/52). After 4 revision surgeries, the perforation was closed in 88.5% of the cases (46/52). Success rates for Group A and Group B were 90.0% and 86.4%, respectively (*p* = 0.689). The success rates in idiopathic and iatrogenic cases were 93.3% and 86.5%, respectively (*p* = 0.659).

**Conclusion:**

This study showed that the success rate of sandwich graft technique was higher in medium-sized perforations than large-sized ones and in idiopathic perforations compared to iatrogenic ones, but the latter rate was not statistically significant. This demonstrated that perforation size was not as important in the sandwich graft technique as in flap techniques.

## Introduction

Nasal septal perforation leads to unpleasant symptoms like nasal obstruction, crusting, whistling, epistaxis, and postnasal drip.^1^ Perforations might occur as a result of surgical operations, trauma, inflammatory diseases as well as intranasal drug abuse.^2^ There are many techniques described to close the nasal septal perforations. Most of these techniques are surgically possible when closing small perforations. Closure of medium and large size perforation however might be a more difficult task. The most commonly used technique with intranasal mucosal advancement flaps.^3^, ^4^ (hereinafter referred to as flap technique). In flap technique, we need to prepare flaps bilaterally, which generally requires wide dissection, especially for medium and large perforations. In this technique, the perforation is closed primarily by advancing the prepared mucosal flaps. Flap preparation is the most challenging step, especially for the patients with previous surgical history, and any problem that occurs during this step might directly influence the surgical outcome.

In another technique that can be used to close nasal septal perforations, an interposition graft only is placed between septal mucosa and fixed there. In this technique, intraoperative mucosal closure is not performed. As in the technique we used in this study, the interpositional graft can be prepared as a sandwich graft (hereinafter referred to as sandwich graft technique). The graft forms the basis of the closure process and the mechanism that enables perforation to close is the regeneration capacity of the mucosa. Therefore, a surface on which mucosa can proliferate is interposed between mucosal flaps.^5^

In the present study, we aimed to demonstrate the way sandwich graft technique (SGT) is applied and present results/outcome of SGT in medium and large perforations.

## Methods

### Study design and methods

The cases operated using the SGT at the Department of Otorhinolaryngology at Hacettepe University from January 2014 to December 2018 were retrospectively reviewed. All operations were performed by one senior author (SO). This study has received Institutional Review Board/Ethics Committee approval from our institution (GO 16/55-26). The cases older than 18 years of age with a followup of at least 6 months, with endoscopic examination findings and questionnaire results, were included in the study. Patients with systemic vasculitis findings and a history of chronic intranasal drug use were excluded from the study. Patients’ demographic data, presenting symptoms, perforation etiologies, endoscopic examination videos, perforation sizes and the graft materials used were retrieved from hospital records.

Subjective symptoms were evaluated with a validated visual analog scale (VAS) performed before and 6 months after surgery. Nasal obstruction, crusting, bleeding, and pain were assessed (0 represented no symptoms and 10 represented maximal symptoms). The sizes of perforations were measured preoperatively and intraoperatively (after the mucosal flaps were prepared). Surgical outcomes were presented according to both perforation etiologies (idiopathic or iatrogenic) and sizes (Group A: < 2 cm, Group B: ≥ 2 cm). According to the perforation dimensions measured intraoperatively, cases with a perforation diameter less than 2 cm were classified as Group A, and cases with a size of 2 cm or more were classified as Group B. The cases where complete closure of perforation was achieved at postoperative 6 month follow up visit were deemed as successful.

### Surgical technique

All surgeries were performed under general anesthesia. We preferred an open rhinoplasty approach for the cases having perforations 2 cm or more. Open rhinoplasty was also preferred for the surgeries in which a caudal septal replacement graft would be used to support the nasal tip, and in the cases for which L-strut reconstruction was planned. In other cases, a closed rhinoplasty technique with hemitransfixion incision was used. First, we prepared bilateral mucoperichondrial/mucoperiosteal flaps.

After the accompanying septal deviation was corrected and L-strut reconstruction was planned, the preferred cartilage and fascia grafts were planned. If nasal septum cartilage was sufficient, it was preferred for the sandwich graft. In cases where septum cartilage was not sufficient, conchal cartilage was used. In cases where septal cartilage and conchal cartilage were used, the temporalis muscle fascia was preferred primarily to wrap the cartilage. In some of the medium size perforations and in all large size perforations, as well as in cases where L strut reconstruction had to be performed, costal cartilage was preferred because too much graft material was needed. When costal cartilage was preferred, we primarily used the anterior rectus abdominis muscle fascia to wrap the cartilage. However, in some cases, we had to obtain fascia from other regions as we could not find enough fascia at that site. Sandwich graft was prepared using the harvested cartilage and fascia grafts. If the rib is preferred as the cartilage source, a segment of 2–3 cm was usually sufficient, and grafts were prepared by the oblique split method (Supplementary material Video 1).^6^

**Figure 1:**
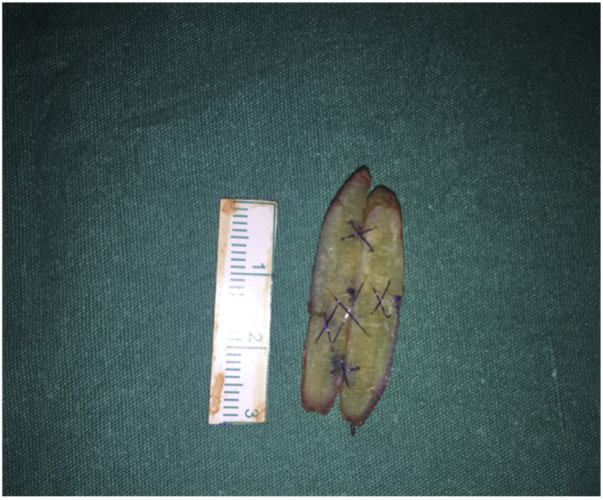
Forming the cartilage block with the costal cartilage grafts.

**Figure 2:**
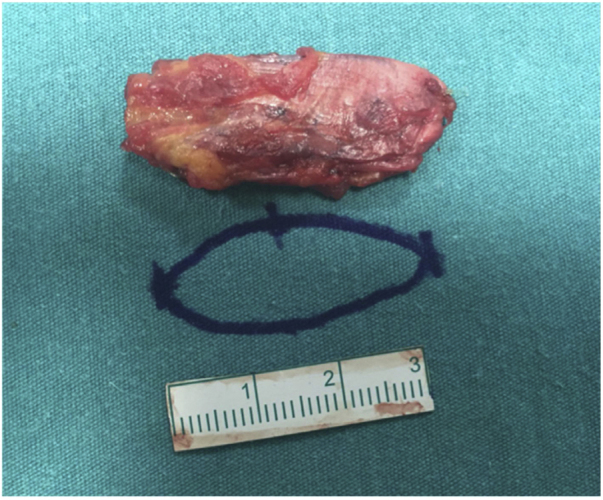
The cartilage block wrapped with fascia.

**Figure 3:**
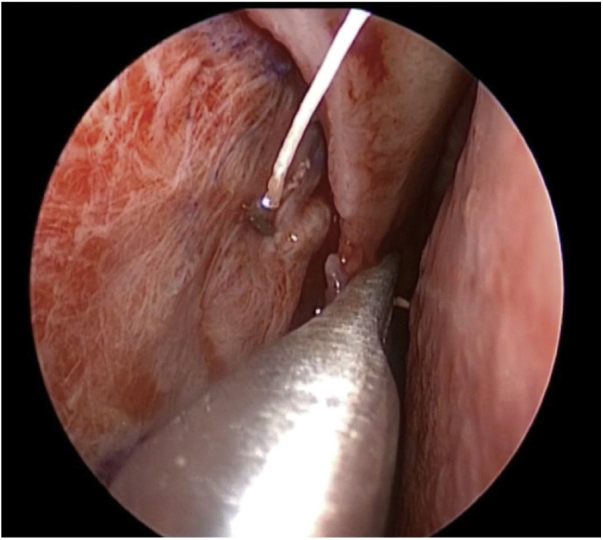
Suturing the mucosal flap to the fascia covering the sandwich graft.

A template of the perforation was drawn on the back table. If a single cartilage graft was not sufficient to fill this template, a cartilage block was formed by sewing them side-by-side (Fig. 1). The cartilage block was put within that fascia and the fascia was sutured with 5/0 vicryl suture around the block to cover it completely (Fig. 2). The formed sandwich graft was put between the mucopericondrial flaps (Supplementary material Video 1). 6/0 vicryl suture with an 11 mm needle was used to suture the fascia of the sandwich graft to the mucosal margins on both sides of the nasal cavity. Suturing should be continued to ensure that the graft and mucopericondrial flap contact each other at all points (Fig. 3) (Supplementary material Video 1). Doyle nasal silicone splint with airway was placed in both sides of the nasal cavity, fixed to the columella with a non-absorbable transseptal suture, and left there for 2–3 weeks depending on the size of the perforation. We recommended the use of postoperative systemic antibiotic (amoxicillin/clavulanate potassium 1 g tablets bid po) for 10 days. Nasal irrigation and moisturizing with topical gel or cream were recommended just after the operation until healing was complete.

Patients were examined in the first postoperative week to remove transcolumellar sutures if present and check the presence of any problem at the donor sites. A second visit was performed for the removal of the silicone splint and endoscopic examination of the nasal cavity. Evaluation of the surgical success and symptom surveys were conducted during the 6^th^ month followup visit.

### Statistical analyses

Data analyses were performed by using SPSS for Windows, version 22.0 (SPSS Inc., Chicago, IL, United States). Whether the distribution of continuous variables was normal or not was determined by Kolmogorov Smirnov test. Levene’s test was used for the evaluation of homogeneity of variances. Unless specified otherwise, continuous data were described as mean ± SD (Standard Deviation) for normal distributions, and median (minimum and maximum) for skewed distributions. Categorical data were described as number of cases.

Quantitative data were evaluated in percentage. For the comparison of the groups, student *t*-test^#^ was used for the normally distributed independent data comprising 2 groups, Mann Whitney u-test^β^ was used for the nonnormally distributed independent data, and Chi-Square or Fisher exact test^Φ^ was used for categorical data. In addition, the differences between non-normally distributed variables of two dependent groups were analyzed by Wilcoxon sign rank test. Significance was taken *p* < 0.05.

## Results

We reviewed 52 patients who were operated with the SGT and followed for an average of 36 months ± 11.75 (mean ± SD). In total 56 surgical operations were performed using this technique from January 2014 to December 2018. Of the 52 patients, 19 were female and 33 were male, and their mean age was 38.5 years ± 11.75 (mean ± SD). None of the patients had a history of topical drug use or an underlying inflammatory or rheumatic disease. The average perforation sizes measured preoperatively and intraoperatively were found to be 16.5 mm ± 6.27 (mean ± SD) and 19.02 mm ± 7.00 (mean ± SD) in diameter, respectively. Considering the etiology of perforations, 37 (71.15%) of the cases had a history of nasal surgery and were considered iatrogenic. In the other 15 (28.84%) cases, no reason for the perforation was found and it was deemed idiopathic. While the open rhinoplasty approach was preferred in 40 cases (71.4%), a closed approach (28.6%) was used in 16 cases.

The success rate after initial surgeries was 84.6% (44/52). In 3 cases that were deemed unsuccessful, it was observed that the sandwich graft was alive, but a perforation remained as a slit. In the other 5 cases, the grafts were necrotic and there was no change in the perforation dimensions. Revision surgery was performed in 4 cases and 2 of them were successful. After revision surgeries, the total success rate became 88.5% (46/52).

When the cases were classified according to their perforation sizes, there were 30 cases in group A (< 2 cm) and 22 cases in group B (≥ 2 cm). The mean intraoperative perforation dimensions measured were 14.1 mm ± 3.00 (mean ± SD) in group A and 25.7 mm ± 5.16 (mean ± SD) in group B. It was seen that the size difference between the groups was statistically significant (t = 9.931, *p* < 0.001). After the first surgeries, surgical success was found in 86.6% (26/30) in group A and 81.8% (18/22) in group B. After revision surgeries, the success rate became 90.0% (27/30) in group A, 86.4% (19/22) in group B. This difference was statistically insignificant (*p* = 0.689).

When the cases were evaluated according to their etiology, it was observed that the intraoperative perforation dimensions of idiopathic (n = 15) and iatrogenic (n = 37) cases were very close to each other (mean ± SD: 16.6 ± 4.88, 20.0 ± 7.48 respectively) (*p* = 0.203). After initial surgeries, the success rates in idiopathic and iatrogenic cases were found as 93.3% and 81%, respectively (*p* = 0.267). Surgical success rates were 93.3% in the idiopathic group and 86.5% in the iatrogenic group after revision surgeries (*p* = 0.659). Although the success rate in the idiopathic cases was higher than that of iatrogenic cases, this difference was not statistically significant.

**Figure 4:**
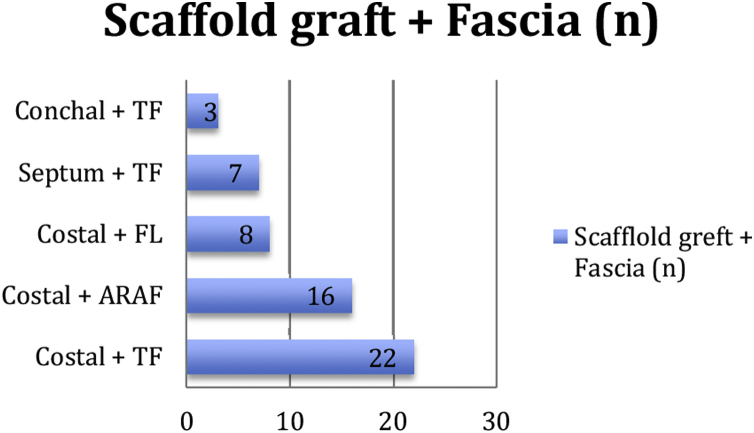
Sandwich graft compositions. TF, Temporal Fascia; FL, Fascia Lata; ARAF, Anterior Rectus Abdominus Fascia.

The graft materials we used to prepare the sandwich graft are given in Fig. 4. It was seen that costal cartilage was preferred as the scaffold graft for the majority of the cases (82.6%). As the fascia graft, temporal fascia was used in 51.9% of the surgical operations. Costal cartilage harvesting both prolongs the surgical time and leadsto additional donor site morbidity. While pain in the donor area was observed in most of the cases, none of them developed pneumothorax or hematoma.

The most common symptoms voiced by patients were nasal obstruction and crusting. The scores of the symptom’s severity before and after the surgery evaluated with VAS are given in Table 1. It was seen that significant improvement was achieved in all symptoms (*p* < 0.001).

**Table 1:** Pre-operative and post-operative VAS scores (Wilcoxon test).

Symptoms	n	Preop score Median (min–max)	Postop score Median (min–max)	Z test	*p*
Obstruction	52	7 (3–10)	3 (1–5)	−6.241	<0.001
Crusting	52	6.5 (3–10)	2.5 (0–5)	−6.178	<0.001
Bleeding	52	3.5 (1–9)	2 (0–8)	−5.487	<0.001
Pain	52	2 (0–8)	1 (0–5)	−3.882	<0.001

VAS, visual analog scale.

## Discussion

Septal perforation surgery is a challenging operation no matter which technique is used. This study, performed in 52 patients with an average follow-up time of 3 years, showed that the SGT was effective in correcting medium and large nasal septal perforations. Perhaps the most striking finding of this study is that SGT is an effective method independent of perforation size.

More valuable data would have been obtained if this work had been designed as a comparative and prospective study. Although the data presented in this study involved a single surgical technique, the sandwich grafts used had different compositions. Therefore, no comparison could be made as to which graft or graft composition was more successful. In fact, there is no specific graft which has been particularly recommended in the literature or reported to be more successful than the others. The general view is that the graft materials used do not change the result.^1^ In addition objective assessment with acoustic rhinometry and measurements of mucociliary activity, besides additional subjective assessment, could be beneficial. This study is limited to VAS (psychosomatic dimension) to obtain data on QoL. Other dimensions of Qol: more specific functional dimension (daily life activities), social dimension and psychological-emotional dimensions (anxiety, depression) were not studied.

The results given in the literature on the flap technique, which is the most frequently used one in perforation surgery, are very successful. In their study, Pedrosa et al. reviewed cases operated with flap technique (57% of them had a perforation size between 1–2 cm) and reported 97% successful closure rate.^4^ In another study, Ribeiro and Silva reported that only in 3 of the cases (ranging 1 to 3.5 cm in diameter) they failed to achieve complete closure.^7^ However, the flap technique requires a larger dissection SGT, and this is much more difficult, especially in patients with a previous history of surgery. Any problem that occurs during this step might directly influence the surgical outcome. The most important criterion for the flap to be successful is to ensure that there is wound closure without tension.^8^ However, if there is any tension it is more reasonable to apply SGT and affix the interposition graft to the mucosal edges with sutures, rather than sewing the flaps in this manner. The preparation stage of mucosal flaps takes shorter time in the sandwich technique than in the flap technique. However, when we also consider the stage of graft preparation, although there was not any data about the surgical times, we think that both techniques do not differ in terms of surgical times.

There are studies in the literature that use a similar technique to that we use in our study and obtain similar results. The features of the studies using interposition grafts are given in Table 2. Kaya et al. used 3-layer grafts prepared with conchal cartilage and temporal fascia in order to repair 22 cases of perforations smaller than 2 cm and reported a success rate of 86.3%.^9^ Chen et al. conducted a study by using interposition grafts for 13 cases with perforation sizes of 1–2 cm in diameter and reported a success rate of 92.3%.^5^

**Table 2:** Some features of studies using sandwich graft technique.

Authors	Feng-Hong Chen et al.	Kaya et al.	Ozer et al.
Technique	Endoscopic sandwich technique	3-layer interlocking method	Sandwich graft
Graft composition	autologous septal cartilage or bone + quadriceps fascia + middle turbinate mucosa (> 2 cm)	Conchal cartilage + temporal fascia	Cartilage (costa-concha-septum) + fascia (temporal fascia-ARAF-fascia lata)
Perforation size	1–2 cm	2 cm > mean: 17.4-mm	Medium and large mean: 19.2-mm
Patients (n)	13	22	52
Attachment/ Stabilization	Biological glue	Stapler and sutures	Sutures to the edge of flaps
	Gelfoam gauze	Without nasal splint	Nasal splint
	Expandable sponge packing		
Closure rate	92.3%	86.3%	88.5%

ARAF, Anterior Rectus Abdominus Fascia.

In our study, a different variation of the same technique was used, and parallel with the findings of the above-mentioned studies, similar success rates were achieved in cases with larger perforations and long-term followup. In our SGT, we preferred to use sutures between the sandwich graft and mucosal flaps. Feng-Hong Chen et al. used fibrin sealant after interposing the interpositional graft, and put expandable sponges to the nasal passage.^5^ The use of fibrin sealants probably makes the procedure easier; however, since it causes extra cost and we had doubts as to its durability during the healing process, we elected not to use it.

The interposition grafts are not always used in the flap techniques. However, although not statistically significant, the success rate was found to be high when used.^10^ In most of the techniques where mucosa closure is not performed, interposition graft is prepared as multi-layer. The cartilage block that we use in the sandwich graft helps to unfold the fascia graft to create a flat floor between the flaps. We think that having a flat surface between the flaps is vital for healthy mucosal healing.

Unlike the flap technique, mucous membranes are not moved to close the defect in SGT. Intense crusting may be expected because there is no mucosal closure. While it was stated in Chen's study that the crusts were cleaned regularly, no information was given about how long it took to resolve.^5^ In our practice, the silicone splints were kept for 3 weeks. We did not see any crusting after the splint has been removed. Chen used expandable sponges for 3 days after surgery.^5^ Ercan Kaya et al. stated that they did not use any splint in their work however, they did not mention how much crusting this caused.^9^

In studies using flap techniques, the success rate in large perforations was found to be significantly lower than in small ones. Kim and Rhee reported that the success rate in small perforations was 93%, while it diminished to 78% in large perforations.^10^ Likewise, Kridel and Delaney achieved full closure in 96.7% of the perforations with diameters smaller than 1.5 cm, while the success rate in perforations with diameters greater than 1.5 cm was found to be 71.4%.^11^ In our study, the success rate in medium sized perforations was 90.0%, while it was 86.4% in larger ones. It was seen that there is no significant difference in the success rates achieved in both groups, although their preoperative perforation sizes were significantly different. This finding implies that perforation size is not as crucial for the SGT as it is for the flap technique. In other words, perforation size is more influential on success in the cases where flap technique is used for perforation closure.

One of the most difficult steps of nasal septum perforation surgery is the elevation of mucoperichondrial flaps. In patients who have undergone surgery before, finding the correct plane and preparing flaps are especially difficult in cases with atrophic mucosa. The health of the mucosa prepared in these cases is also a question mark and unhealthy mucosa should be excised at the expense of enlarging the perforation dimensions. Another finding of our study is that a higher success was obtained in idiopathic cases than in iatrogenic ones, although it was not statistically significant. To the best of our knowledge, there is no study in the literature in which the effect of etiologic causes on success was investigated and compared.

In SGT, a successful perforation closure depends on the mucosal regeneration occurring on the graft interposed between the flaps. Hence, the graft should be kept in its place until the mucosal healing process is complete. The preferred graft should remain viable during this time, as it takes longer to achieve mucosal closure in large perforations. In our study, it was seen that the graft we prepared remained vital enough to allow the closure of even the large perforations. We believe instead of preparing intranasal flaps, using a sandwich graft for all sizes of perforations after elevating the mucosa at the edges of the perforation will make the surgery easier.

During the healing process of SGT, mucosa regenerates on the interposed fascia and epithelializes perforation site; long vitality of sandwich graft may be playing a role in successful closure of perforation. We think that suturing provided the mucosal flap and sandwich graft intimate contact, and it is the most important component of this SGT. However, suturing inside the nose is technically difficult. We need new instruments that will help us tie the sutures more easily and quickly in order to accelerate and ease the septal perforation surgery. Further studies are to be conducted in this regard and will contribute to ensuring that septal perforation surgery is technically no longer a problem.

## Conclusion

Using SGT for closure of medium and large size nasal septal perforations leads to high closure rate irrespective of the size of perforation. This demonstrates that perforation size is not as important in SGT as the choice of the flap technique. Sandwich graft technique achieved the high success rate for iatrogenic and idiopathic septal perforations.

## Funding

This research did not receive any specific grant from funding agencies in the public, commercial, or not-for-profit sectors.

## Conflicts of interest

The authors declare no conflicts of interest.

## Appendix A Supplementary data

Video S1 A surgical video of a 45 year-old male with a 2 cm perforation considered as idiopathic is given. First, mucosal flaps are prepared and then sandwich graft is prepared using with costal cartilage and temporal fascia. Suture repair was done between mucosal flaps and sandwich graft. At the end of the video, the control endoscopic examination record, which is done at the 6^th^ month after surgery, is given.
